# Micropenis in Children and Adolescents: A Narrative Review

**DOI:** 10.1590/S1677-5538.IBJU.2025.0648

**Published:** 2025-12-20

**Authors:** Edson da Silva Salvador, Luciano Alves Favorito

**Affiliations:** 1 Universidade do Estado do Rio de Janeiro Hospital Universitário Pedro Ernesto Departamento de Urologia Rio de Janeiro RJ Brasil Departamento de Urologia, Hospital Universitário Pedro Ernesto, Universidade do Estado do Rio de Janeiro - UERJ, Rio de Janeiro, RJ, Brasil; 2 Universidade do Estado do Rio de Janeiro Unidade de Pesquisa Urogenital Rio de Janeiro RJ Brasil Unidade de Pesquisa Urogenital, Universidade do Estado do Rio de Janeiro - UERJ, Rio de Janeiro, RJ, Brasil

**Keywords:** Child, Hypogonadism, Testosterone

## Abstract

**Purpose:**

To summarize current evidence on the etiology, diagnostic approach, management strategies, and outcomes of micropenis in children and adolescents.

**Materials and Methods:**

A narrative review was performed using PubMed/MEDLINE (October 2025) with the search terms (Micropenis OR Microphallus OR "Small Penis") AND (Children OR Youth OR Adolescents). From 707 records screened, 36 studies were selected based on methodological quality and relevance to clinical practice.

**Results:**

Micropenis is a clinical sign frequently associated with underlying endocrinopathies, particularly Congenital Hypogonadotropic Hypogonadism (CHH). Accurate diagnosis relies on standardized Stretched Penile Length (SPL) assessment, recently optimized by the Stretched Penile Length INdicator Technique (SPLINT). Use of population-specific SPL nomograms is critical for diagnostic reliability. Testosterone therapy remains the primary treatment modality and demonstrates greatest efficacy in early infancy, promoting significant penile growth and generally favorable functional outcomes. Spontaneous catch-up growth during puberty has been reported in select cases. Current evidence supporting surgical interventions in children and adolescents is limited, heterogeneous, and associated with inconsistent long-term results; thus, surgery should not be considered first-line therapy. High-quality long-term outcome data and randomized placebo-controlled trials are lacking.

**Conclusions:**

Standardized SPL measurement and appropriate nomogram use are essential for accurate diagnosis. Early hormonal therapy, especially in CHH-associated micropenis, appears to yield optimal functional and psychosocial outcomes. Expectant management may be appropriate in selected clinical scenarios. Surgical techniques remain controversial, with insufficient evidence to recommend routine use. Further well-designed prospective studies, including randomized placebo-controlled trials, are needed to define long-term outcomes and guide clinical decision-making.

## INTRODUCTION

Micropenis is a clinical diagnosis characterized by a structurally normal, albeit small, penis (1). The condition is defined by a Stretched Penile Length (SPL) that falls 2.5 standard deviations (SD) or more below the mean in a chart for a patient's age and level of sexual development (2). The identification of micropenis in infancy or childhood is of paramount importance, as it is frequently the presenting sign of a significant underlying congenital or acquired endocrinopathy (3, 4). The clinical relevance of micropenis extends beyond its physical manifestation. The diagnosis can cause considerable anxiety for parents, significant psychosocial distress, body image issues, self-esteem problems, concerns about future sexual function and loss of Quality of Life (5). Historically, the management of micropenis has been a subject of controversy, with past recommendations even including the now-obsolete consideration of gender reassignment for the most severe cases (6). However, cumulative evidence from follow-up studies, albeit with persisting knowledge gaps, has considerably advanced our understanding, especially in the context of hormonal therapy. (7). This narrative review aims to provide a comprehensive overview of the current state of knowledge regarding micropenis in the pediatric and adolescent population. The relevance of the topic, the diagnostic process with a comparison of the principal growth charts used globally, the mainstays of treatment, and the reported outcomes based on contemporary scientific evidence will be covered.

## MATERIALS AND METHODS

A comprehensive literature search was conducted on PubMed/MEDLINE in October 2025. The search strategy employed Medical Subject Headings (MeSH) and free-text terms: (Micropenis OR Microphallus OR "Small Penis") AND (Children OR youth OR adolescents), unrestricted by date or language, with a focus on articles published in English.

**Figure 1:**
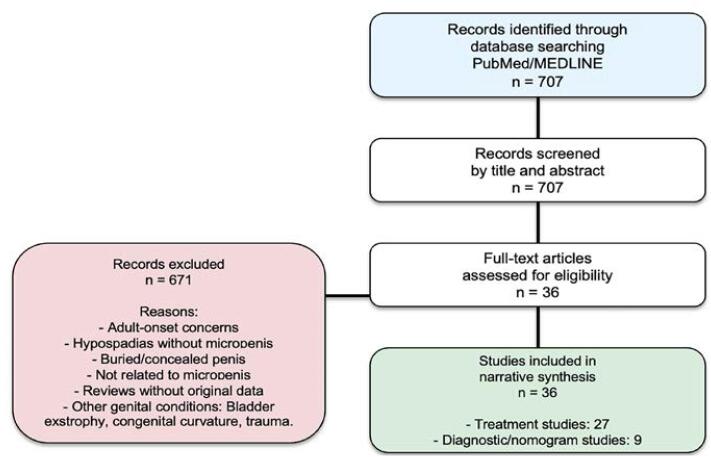
PRISMA flowchart of the study selection process.

Initial results were screened by title and abstract for pediatric/adolescent relevance. Inclusion criteria: articles discussing etiology, diagnosis, treatment, or outcomes. Exclusion criteria: adult-onset concerns, hypospadias, epispadias, bladder exstrophy, buried/concealed penis unrelated to shaft length deficiency or other genital abnormalities. A total of 707 articles were identified. Of these, 36 key articles were selected for this review based on their relevance, study design, and contribution, with a focus on studies reporting penile growth charts and treatment results. Reviews and articles that did not mention diagnosis or treatment results were also excluded (Figure-1).

## RESULTS

### a. Relevance of the Topic in Pediatric Urology

Micropenis is a relevant topic in pediatric urology and endocrinology primarily because it serves as a critical physical marker for underlying systemic diseases. The precise global prevalence is unknown, but data suggest an incidence of approximately 1 in 300 male births, with a reported incidence in North America of approximately 1.5 per 10,000 male newborns (8). The condition is most often a consequence of insufficient androgen stimulation for penile growth during a critical window of fetal development, specifically from 12 weeks of gestation through the postnatal "mini-puberty" in the first six months of life.

The most common underlying known cause is Congenital Hypogonadotropic Hypogonadism (CHH), a failure of the testosterone axis (9). Furthermore, micropenis can be a feature of numerous genetic syndromes, such as Prader-Willi, Kallmann, and Klinefelter syndrome, making its recognition a key step in a broader diagnostic workup (10). A full medical evaluation is essential not only to address the penile size itself but also to diagnose and manage potentially life-threatening associated conditions, such as hypoglycemia in cases of panhypopituitarism (Table-1).

**Table 1:** Surgical Approaches for Micropenis.

Reference Number and Year	Technique Description	Outcomes & Complications	Author's Remarks / Goals
Hinman 1971 (33)	**Two-Stage Elongation and Burial: Stage 1:** Corporal bodies are dissected to their base for maximal length and then buried in subcutaneous 2. **Stage 2 (3-4 months later):** The penis is liberated, and skin coverage with thick scrotal flaps.	Outcomes not quantitatively reported.	Aims to allow for vascular adaptation and shaft elongation before providing skin coverage.
Gilbert et al. 1993 (34)	**One-Stage Microsurgical Free Flap Phalloplasty (Radial Forearm):** Radial forearm free flap to create a neophallus. Vascular anastomoses are made to epigastric vessels, and nerve coaptation is performed with the pudendal nerve for sensation.	**Success Rate:** 91%. **Complications:** Urethral fistulas (5 cases), strictures (3 cases). **Sensory Outcomes:** All patients with nerve coaptation regained protective and erogenous sensation.	Goals are to achieve voiding while standing, preserve sensation, create a phallus suitable for a prosthesis.
Perović et al. 1995 (35)	**Extended Pedicle Island Groin Flap:** A flap from the groin and lower abdomen, based on superficial iliac and epigastric vessels, is used. It is designed in three parts to create a neourethra and neophallus.	All patients achieved a cosmetically and functionally satisfactory neophallus. **Complications:** Partial flap necrosis (2 cases), urethral fistula (2 cases), anastomotic stenosis (1 case). **Sensitivity:** Generally mild to moderate.	The technique aims to create a complete neophallus with a neourethra in a single stage, with glans sculpting performed later.

### b. Diagnosis and Comparison of Growth Charts

**Figure 2:**
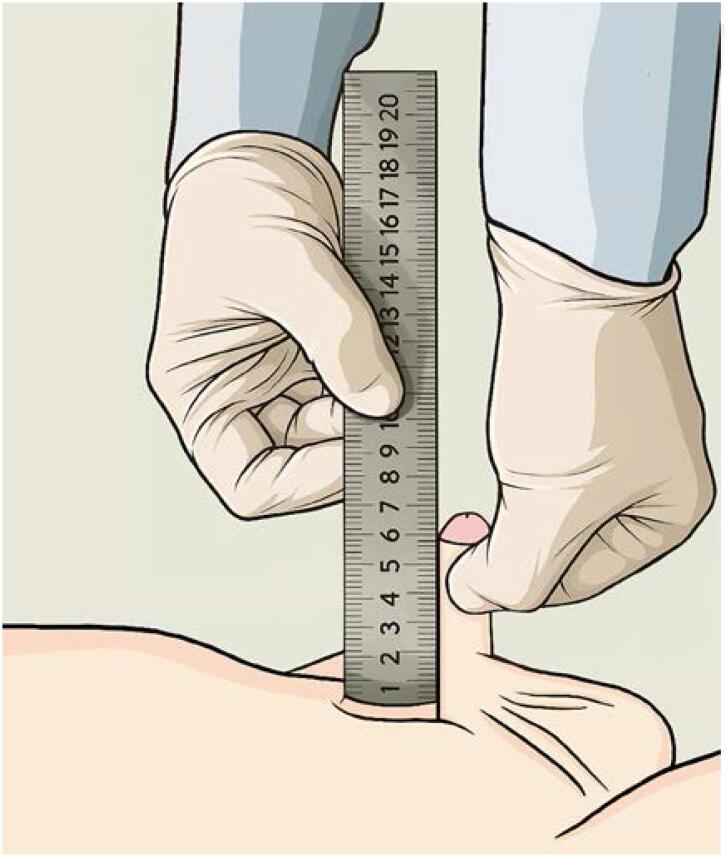
SPLINT (Stretched Penile Length INdicator Technique). Note the private ambient room, supine position, foreskin retraction (for those who doesn't have phimosis), use of rigid ruler with zero-error correction and the compression over the suprapubic fat. The penis is stretched vertically to the point of resistance without causing discomfort. At least two (preferably three) measurements are obtained to ensure reproducibility.

The diagnosis of micropenis is clinical, based on an accurate measurement of SPL. A 2024 systematic review (11) highlighted significant heterogeneity in measurement methodologies across 145 studies. This review identified several factors that influence the accuracy of SPL measurements. To address these inconsistencies, the authors proposed a standardized protocol named the Stretched Penile Length INdicator Technique (SPLINT) – (Figure-2).

Figure Source: The Author.

A cornerstone of diagnosis is the use of penile length nomograms. These charts provide the mean and standard deviations for SPL across different ages. However, there is a significant finding in the literature about the well-documented variation in penile size across different ethnic and geographic populations. This has led to the development of numerous population-specific nomograms. A comparison of the most widely used charts is presented in Table-2.

**Table 2:** Review of SPL Nomograms.

Reference Number	Year	Population	Key Characteristics
Teckchandani and N, Bajpai (12)	2014	Indian	200 patients (0-10y); two measures in supine position by the same observer. Excluded endocrine and genetic syndromes.
Ishii et al. (13)	2015	Japanese	1628 patients (0-7y); multicentric cohort. Absence of genital anomalies, endocrine disorders or major malformations.
Gul et al. (14)	2021	Turkish	948 healthy, uncircumcised boys; single center, one examiner. Excluded genital/congenital abnormalities.
Ibrahim et al. (15)	2023	Egyptian	1500 prepubertal patients (5-9y); single center, single observer. Excluded chronic illness, abnormal growth, and uncircumcised boys.
Krämmer et al. (16)	2025	Brazilian	140 Preterm male newborns; measures within 72h of life, repeated weekly. Single examiner.
Gabrich et al. (17)	2007	Brazilian	2,010 participants (0-18y); heterogeneous cohort. Three examiners. Dual classification by age and Tanner stage.
Wang et al. (18)	2018	Chinese	2,974 healthy urban boys (0-17y); two trained examiners.
Tomova et al. (19)	2010	Bulgarian	6,200 healthy white boys (0-19y); single endocrinologist. Included testicular volume and penile circumference.

As the table illustrates, there are just few anthropometric pediatric populations sampling around the World. Therefore, clinicians should use the most relevant, up-to-date, and population-specific data available to accurately diagnose micropenis and local data record charts are undoubtedly the best way to diagnosis micropenis.

### c. Treatment

The initial objectives of micropenis management are counseling, investigation of underlying endocrinological causes (as often as possible) and hormonal therapy, with the goal to stimulate penile growth to achieve a length that is within the normal range for age. Surgical options are reserved for cases where hormonal therapy fails to achieve adequate penile length, or in the presence of anatomical abnormalities.

#### c.1. Hormonal Therapy

The most widely accepted and effective treatment for micropenis, which can be particularly effective in cases of CHH, is hormonal therapy, but some patients may not reach normal adult penile size, especially in cases of severe hypogonadotropic hypogonadism (20). Monitoring for side effects such as premature virilization and elevated serum testosterone is recommended, particularly with topical therapy. There are no large, placebo controlled, long-term studies and evidence-based guidelines directly addressing testosterone therapy for micropenis, and further research is needed to optimize treatment timing and assess long-term outcomes.

According to medical literature, the optimal timing for testosterone therapy to achieve the best response in penile growth for patients with micropenis is during infancy or early childhood, including the period of mini puberty. Early initiation of therapy is associated with greater penile growth, and initial penile dimensions – particularly glans width – are strong predictors of response (21-23). Table-3 summarizes the main study results with testosterone for micropenis.

**Table 3:** Hormonal Management of Micropenis.

Reference Number	Study type, Substance(s), Patient Cohort	Posology	Key Outcomes & Remarks
Ishii et al. 2004 (24)	Prospective, Testosterone Enanthate (TE), 53 Japanese prepubertal boys.	25mg IM every 4 weeks, up to 4 times.	**Effective:** Median SPL increment of 0.6cm, independent of age or gene polymorphisms.
Karrou et al. 2023 (25)	Prospective, Transdermal Dihydrotestosterone (DHT) vs. TE, 49 boys without hypogonadism or genetic syndromes.	**DHT:** 5mg daily for 5 weeks (renewed 1-2 times). **TE:** 50mg IM monthly (renewed once).	**DHT Superiority:** Mean growth DHT +2.37 cm vs. TE +1.82 cm (p=0.008). **No Side Effects Critique:** Small sample size, no genetic testing.
Bin-Abbas et al. 1999 (26)	Retrospective, Testosterone Enanthate (TE), 8 males (18-27y) with CHH.	25-50mg IM every 4 weeks for 3 months (1-2 courses), then dose increased to adult regimen.	**Long-Term Success:** No significant difference between early (infancy) vs. late (childhood) treatment.
Nerli et al. 2013 (27)	Retrospective, TE vs. hCG, 25 boys with isolated non-syndromic micropenis.	**TE (<11y):** 25mg IM monthly for 3 months. **hCG (>11y):** 1,500-2,000 IU IM weekly for 6 weeks.	**Significant Growth:** >100% increase in SPL in both groups. No adverse effects reported.
Becker et al. 2016 (28)	Retrospective, hCG, 20 patients with CHH.	1,500-2,000 IU IM, 3x/week for 8 weeks.	**Effective for IHH:** Mean SPL increased 2.31 cm. Safe and well-tolerated.
Arisaka et al. 2001 (29)	Prospective, Topical Testosterone, 50 prepubertal boys (5mo-8y).	5% cream (10mg) applied daily for 30 days.	**Significant Growth:** Mean SPL increased ~44%,. **Minimal Side Effects:** Mild, transient local hyperpigmentation/eczema. No skeletal effects.
Xu et al. 2017 (30)	Open Prospective, DHT Gel, 23 boys (9mo-11y) with normal karyotype.	2.5% gel (0.1-0.2 mg/kg/day) applied daily for up to 6 months.	**High Success Rate:** 61% achieved normal SPL (> −2.5 SD). 26% clinically improved. **Safe:** No bone age acceleration or systemic side effects.

#### c.2 Surgical Treatment

Surgical intervention, as documented in medical literature, is not a first-line treatment for micropenis in children. Surgical techniques are complex and include procedures like the release of the suspensory ligament (31) and neo phalloplasty. The outcomes of these surgeries in the pediatric population are not well-documented, and they carry significant risks, making hormonal therapy the preferred initial approach. The Brazilian Federal Medical Council, under Resolution 1.478/1997, considers penile lengthening surgery for sexual dysfunction to be experimental and restricts its performance to rigorously controlled human research protocols (32).

## DISCUSSION

Micropenis is clinically significant because it frequently reflects underlying disruptions in androgen endocrinologic axis, with Congenital Hypogonadotropic Hypogonadism (CHH) being the most common identifiable etiology. Its presence may also indicate broader syndromic conditions, emphasizing the role of micropenis as an early diagnostic marker within multidisciplinary evaluations (36).

Accurate diagnosis depends on correct use of standardized Stretched Penile Length (SPL) measurement protocols. The literature demonstrates substantial heterogeneity in measurement techniques, increasing the risk of misclassification. The recently proposed Stretched Penile Length Indicator Technique (SPLINT) offers a reproducible method designed to mitigate these discrepancies, although further validation across diverse populations is required. Given the documented ethnic and regional variability in penile length, the use of population-specific nomograms remains essential for diagnostic reliability.

Testosterone therapy remains the most effective and widely accepted treatment. Studies consistently demonstrate significant penile growth, particularly when initiated in infancy or early childhood, corresponding to periods of heightened androgen sensitivity. While short-term outcomes are favorable, long-term data are limited, and randomized placebo-controlled trials are lacking. Factors such as baseline penile size may influence treatment response, but standardized predictive markers have not yet been established.

Emerging evidence suggests that many untreated patients may achieve normalization of penile size during puberty, supporting expectant management in selected cases. However, methodological limitations - particularly high attrition rates - restrict the generalizability of this approach. Any expectant strategy must be individualized and accompanied by structured clinical and psychosocial follow-up.

Surgical management remains controversial. The available evidence is scarce, heterogeneous, and limited by small cohorts and inconsistent outcome reporting. Procedures such as suspensory ligament release or phalloplasty are reserved for exceptional situations and should not be considered first-line interventions.

Significant knowledge gaps persist, including the optimal timing and duration of hormonal therapy, long-term functional and psychosocial outcomes, and predictors of spontaneous pubertal growth. Future progress will depend on well-designed prospective studies capable of addressing these limitations.

## CONCLUSIONS

The management of micropenis in children and adolescents relies fundamentally on accurate diagnosis using standardized SPL measurement techniques and population-specific nomograms. Hormonal therapy, particularly in cases related to CHH, remains the cornerstone of treatment and generally yields favorable functional and psychosocial outcomes when initiated early. Emerging evidence suggests that expectant management may be appropriate in select individuals due to the potential for spontaneous pubertal catch-up growth, although further validation is required. Surgical interventions lack robust evidence, show inconsistent outcomes and high morbidity, and should not be considered first-line therapy in this population. High-quality prospective studies, including randomized placebo-controlled trials, are needed to define long-term outcomes, refine patient selection, and guide evidence-based management strategies.

## Data Availability

All data generated or analysed during this study are included in this published article

## ABBREVIATIONS

BMI: = Body Mass Index
CHH: = Congenital Hypogonadotropic Hypogonadism
DHT: = Dihydrotestosterone
GnRH: = Gonadotropin-Releasing Hormone
HPG: = Hypothalamic-Pituitary-Gonadal
LH: = Luteinizing Hormone
FSH: = Follicle-Stimulating Hormone
SD: = Standard Deviation
SPL: = Stretched Penile Length
SPLINT: = Stretched Penile Length INdicator Technique
TE: = Testosterone Enanthate
